# Optical Strain Gauge Prototype Based on a High Sensitivity Balloon-like Interferometer and Additive Manufacturing †

**DOI:** 10.3390/s22197652

**Published:** 2022-10-09

**Authors:** Victor H. R. Cardoso, Paulo Caldas, Maria Thereza R. Giraldi, Orlando Frazão, João C. W. Albuquerque Costa, José Luís Santos

**Affiliations:** 1 Applied Electromagnetism Laboratory, Federal University of Pará, Rua Augusto Corrêa, 01, Belém 66075-110, Brazil; 2 Institute for Systems and Computer Engineering, Technology and Science, Rua do Campo Alegre, 687, 4169-007 Porto, Portugal; 3 Polytechnic Institute of Viana do Castelo, Rua Escola Industrial e Comercial de Nun’Álvares, 4900-347 Viana do Castelo, Portugal; 4 Laboratory of Photonics, Military Institute of Engineering, Praça Gen. Tibúrcio, 80, Rio de Janeiro 22290-270, Brazil; 5 Department of Physics and Astronomy, Faculty of Sciences of University of Porto, Rua do Campo Alegre, 687, 4169-007 Porto, Portugal

**Keywords:** strain sensor, balloon-shaped, 3D printer, displacement sensor, diameter measurement

## Abstract

An optical strain gauge based on a balloon-like interferometer structure formed by a bent standard single-mode fiber combined with a 3D printer piece has been presented and demonstrated, which can be used to measure displacement. The interferometer has a simple and compact size, easy fabrication, low cost, and is repeatable. The sensor is based on the interference between the core and cladding modes. This is caused by the fiber’s curvature because when light propagates through the curved balloon-shaped interferometer region, a portion of it will be released from the core limitation and coupled to the cladding. The balloon has an axial displacement as a result of how the artwork was constructed. The sensor head is sandwiched between two cantilevers such that when there is a displacement, the dimension associated with the micro bend is altered. The sensor response as a function of displacement can be determined using wavelength shift or intensity change interrogation techniques. Therefore, this optical strain gauge is a good option for applications where structure displacement needs to be examined. The sensor presents a sensitivity of 55.014 nm for displacement measurements ranging from 0 to 10 mm and a strain sensitivity of 500.13 pm/μϵ.

## 1. Introduction

Traditional strain gauges include compress meters, piezoelectric, demec mechanical, electric resistance strain gauges, and others [1,2]. The approach that is most frequently employed is the electric strain gauge. It is a resistance component whose resistance alters under tension, a metallic strain gauge’s resistance changes when a metallic wire’s molecular structure slightly deforms [3,4]. Electrical strain gauges, however, have a limited capacity for multiplexing, are susceptible to electromagnetic interference, are affected by relative humidity, have a short lifespan, etc. [5]. High-sensitivity, low-cost, and simple-to-use requirements for novel applications have sparked the development of new measuring technologies such as fiber optic techniques and interferometry [2]. Additionally, the development of optical fiber for light transmission enables the inspection of locations inaccessible by conventional techniques, thereby enhancing the adaptability of optical techniques for industrial applications.

Optical strain gauges have been widely investigated in recent decades. In comparison with traditional strain gauges, strain gauges based on optical fiber sensors have several advantages such as high sensitivity, compact size, lightweight, humidity resistance, corrosion resistance, immunity to electromagnetic interference, and the ability for long-distance measurements [6,7,8,9,10]. It is possible to cite various kinds of strain gauges based on optical fiber sensors: Fiber Bragg gratings (FBG) [11,12], in-line Fabry–Perot interferometer (FPI) [13], in-line Mach–Zehnder interferometer (MZI) [14], long-period fiber grating (LPG) [15], and so on. Among the types cited, the FBGs stand out. FBGs are commonly used, fixed using glue, or embedded in the structure [16]. For both cases, the theoretical strain sensitivity is 1.21 pm/μϵ [17]. However, occasionally the noise of the FBG interrogator prevents the sensitivity of the FBG from being qualified for the precise detection of small-amplitude strain [16]. Furthermore, in the fabrication process of the FBG, expensive techniques that involve UV or CO${}_{2}$ lasers are used [6]. On the other hand, the in-line interferometers have advantages over FBG, namely, the simple fabrication process and low cost. However, for some types of in-line interferometers, the sensors have an easy breakability, such as interferometers based on optical splicing.

For many applications, MZI is widely utilized and exhibits high sensitivity. Furthermore, MZI-based optical fiber bending structures have fundamentally straightforward designs and ease of manufacture [18]. A balloon-shaped interferometer can be found between these structures. The balloon-like fiber structure offers a potential remedy for this issue due to its intrinsically simple structure and great sensitivity. The structure can be merely constructed by bending the Single-mode fiber (SMF) into a balloon-like form, eliminating the need for a time-consuming and expensive production process [19]. In this setup, a portion of the light will pass from the core to the cladding layer without being constrained by the core, and interference between the core and cladding modes due to their different lengths will then occur [20,21,22]. Curved optical fiber cross-section sensors based on balloons are capable of scanning wide regions with good measurement reproducibility, ease of use, and compactness [23]. In recent years, sensors based on the balloon-like interferometer with highly sensitivity have been proposed for different measuring parameters, including micro-displacement [21], vector magnetometer [19], refractive index (RI) [22,24], force [20], temperature [23,25], and humidity [26].

In addition to the research performed to obtain optical sensors acting as optical strain gauges, additive manufacturing (AM) has been widely used in conjunction with optical fiber sensors in the last years [27,28]. AM is also known as three-dimensional (3D) printing. By incorporating optical fiber sensors into various AM structural materials, this combination can be utilized to quantify deformation, and fatigue damage, and assess the structural integrity [29,30]. According to recent scientific literature, employing AM with integrated optics opens up new possibilities for sensor production and has sped up and improved the development of analytical technology [31,32]. Analytical procedures may be quickly prototyped and improved thanks to 3D printing, which enables manufacturing that is simply specified in software. Because it is possible to construct a structure in accordance with the properties of the sensor or parameter to be examined, it is regarded as an important inspection technique [27]. As a result, there is considerable potential for the creation of novel integrated systems in photonics and optoelectronics [28]. In addition, AM provides the use of biodegradable and biocompatible materials [33]

The balloon-like interferometers have been widely used as sensors to detect micro-displacement and have a limited dynamic range due to the response of the sensors depending on the variation of the radius of curvature. Thus, in this work, the optical strain gauge based on a balloon-like interferometer combined with AM has been experimentally investigated to improve the dynamic range and sensitivity. A bent standard coated-SMF makes up the sensor structure, which is used for displacement sensing. The core mode and cladding modes produced by the bending SMF are interfered with. The balloon-like structure’s bending diameter changes as a result of the displacement used in the 3D component, changing the effective RI of the core mode and cladding modes. The sensitivity and dynamic range of the sensor are limited by the shape of the balloon itself, as it has a maximum displacement that depends on the length of the balloon. Due to it, we developed a mechanism to improve these parameters. Wavelength shift or intensity change interrogation techniques can be used to determine the sensor response. The proposed sensor suggested in this work has advantages over previously reported fiber sensors, including excellent sensitivity, a straightforward configuration, simple production, and low cost.

This paper is organized as follows: Section 2 introduces the sensor operating principle. Section 3 presents the proposed experimental setup with the balloon-like interferometer formation and characterization. Section 4 contains a description of the findings and their discussion, followed by a comparison of the findings. Section 5, which is the conclusion, reveals how the suggested sensor is applied.

## 2. Principle of Operation

**Figure 1:**
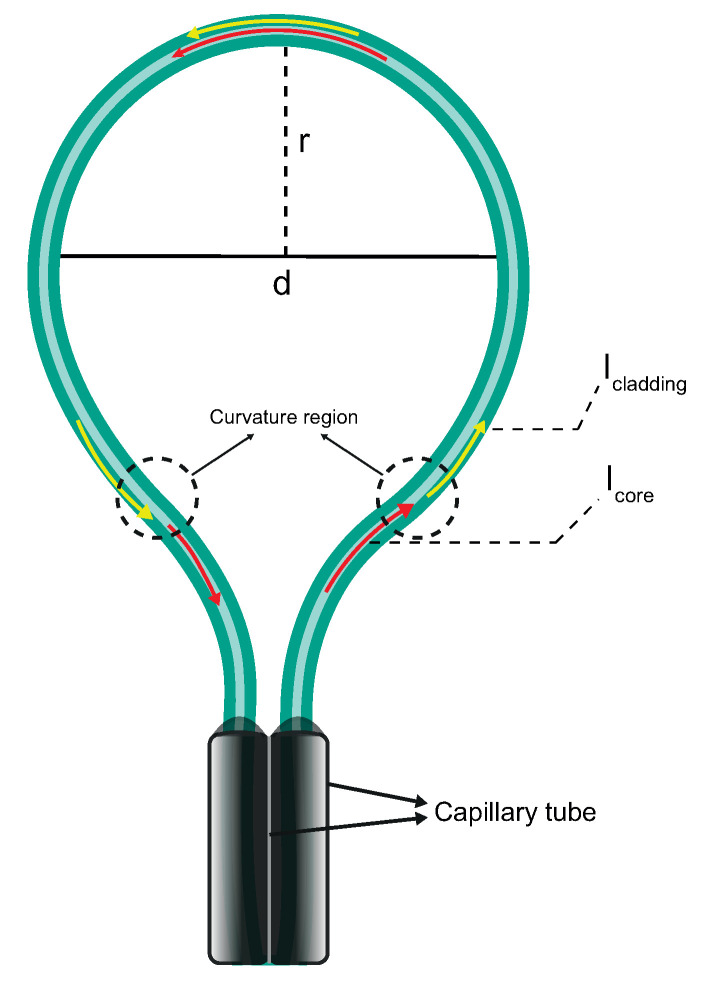
Schematic of the proposed balloon-like interferometer.

In this work, it is proposed and demonstrated an optical strain gauge using a high-sensitivity balloon-like interferometer. The sensor is formed by the bending of the standard SMF. It is a simple, compact, and easy-to-handle sensor. The sensor is described in the schematic diagram of the sensor structure as shown in Figure 1. The bending diameter of the interferometer is defined as *d* and half of the diameter is the bend radius *r*. Modal interference occurs between the core and cladding modes due to the bent SMF when the light propagates in the balloon-shaped structure [20,23,34]. Two capillary tubes are used to hold the fiber, prevent the tangling of the fiber, and maintain the balloon-shaped structure. In addition, this configuration permits adjusting the radius and diameter according to the needs of the application. When a micro-displacement is applied to the interferometer, the bending diameter is changed and that causes the wavelength shift of the modal interference [35].

The basic operating principle of the balloon-like interferometer is based on the interference between the core mode and the cladding modes. This is due to the curvature of the fiber because a part of the light escapes from the core constraint, and penetrates the cladding when the light propagates in the balloon-shaped interferometer curvature section [25], respectively, red and yellow lines shown in Figure 1. A part of their mode power is reflected in the fiber core when the light is coupled to the cladding border. This repeats as light propagates in the curvature region. Thus, a modal interferometer is created due to differences in the effective refractive indexes and the optical path lengths excited by the optical signal carrying the core and cladding modes [20,23,34,36]. The optical power inside the fiber as a function of the distance z can be calculated as give:

(1) $$\frac{I_{t}\left(z\right)}{A_{eff}}=\left[\frac{I_{i}}{A_{eff}}\right]exp\left(-\alpha z\right)$$

where α is the attenuation coefficient. Their different effective refractive indexes and different optical paths lead to modal interference at the output of the device. Since the effective refractive index of the cladding mode depends on the surrounding medium, changes in the surrounding refractive index will affect the propagation velocity, resulting in a spectral shift in the transmission spectrum [20,34,35]. Assuming that just one mode of the cladding is excited in the curvature region of the balloon, the transmission intensity after light passes through the curvature region can be expressed as [20,34,35,37]:

(2) $$I=I_{core}+I_{cladding}+2\sqrt{I_{core}I_{cladding}}\;cos\left(\Delta \phi \right)$$

where $I_{core}$ and $I_{cladding}$ are the intensities of the core mode and cladding modes, respectively. Δϕ is the phase difference between the core mode and cladding modes, which is expressed as:

(3) $$\Delta \phi =\frac{2\pi L(n_{core}-n_{cladding})}{\lambda }=\frac{2\pi \Delta n\;L}{\lambda }$$

where λ is the wavelength, *L* is the effective length of the balloon-shaped interferometer curvature region. $n_{core}$ and $n_{cladding}$ are the effective refractive indexes of the core mode and cladding mode, respectively. Δn is the effective refractive index difference between them. When Δϕ=(2m+1)π, where *m* is an integer, the interference pattern reaches a valley. The resonant wavelength $\lambda_{d}$ can be presented as:

(4) $$\lambda_{d}=\frac{2\Delta n\;L}{2m+1}$$

As demonstrated in Figure 1, the radius (*r*) of a balloon-shaped fiber is equal to the half distance between the centers of two symmetry fibers. In agreement with the stress-optical effect, for the balloon-like interferometer, the refractive index of the outer layers of the bending SMF becomes higher and that of the inner layers becomes lower when compared to the original straight fiber [21]. Consequently, the refractive index profile can be expressed using the equivalent modal refractive index as [35,38,39,40]:

(5) $$n^{\prime }\left(x\right)=n\left(x\right)\left(1+\frac{x}{r}\right)$$

where *r* is the radius of curvature and *x* is the distance perpendicular to the axis of curvature of the SMF. $n^{\prime }\left(x\right)$ and n(x) are the refractive index profiles when the SMF is curved and straight, respectively.

## 3. Balloon-like Interferometer Formation and Experimental Setup

**Figure 2:**
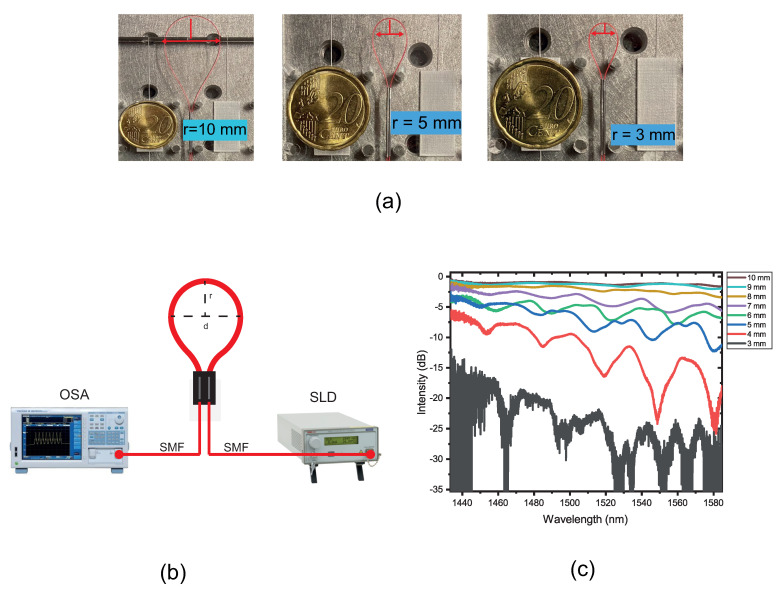
(**a**) Balloon-like interferometer with different lengths and radii of curvature; (**b**) Transmitted spectrum for different values of radius of curvature. (**c**) Proposed setup to balloon-like interferometer formation.

From Equation (4), it is possible to obtain an interferometer with different sensitive lengths and bending radius with different patterns of interference. Firstly, interferometers with different lengths and different radii of curvature, always keeping the balloon-shaped, are investigated, as depicted in Figure 2a. These interferometers were formed using the same SMF. The diameter was reduced as the SMF was pulled back. An optical spectrum analyzer (OSA, AQ6370C, from Yokogawa Inc., Musashino, Tokyo) and an SLD source (S5FC1550S-A2, from Thorlabs Inc., Newton, NJ, USA) were used, as illustrated in Figure 2b.

The transmission spectra for some bending radii, ranging from 3 mm to 10 mm, are shown in Figure 2c. It is possible to observe that as the radius of curvature and the length of the balloon are reduced, the resonant wavelength dip appears and the intensity is decreased. However, for a radius value of 3 mm, it is impossible to examine the spectrum. High attenuation will occur because too much light will leak from the fiber through the cladding. Above 10 mm, there is no resonance interference, so there is no light coupled to the cladding modes and no variation in the transmission spectrum. An optimum interference effect is produced when the bend radius is 4 mm; this results in a clear dip with a resonance depth of roughly −24 dB at 1548 nm.

### Preparation and Characterization Using 3D Piece

**Figure 3:**
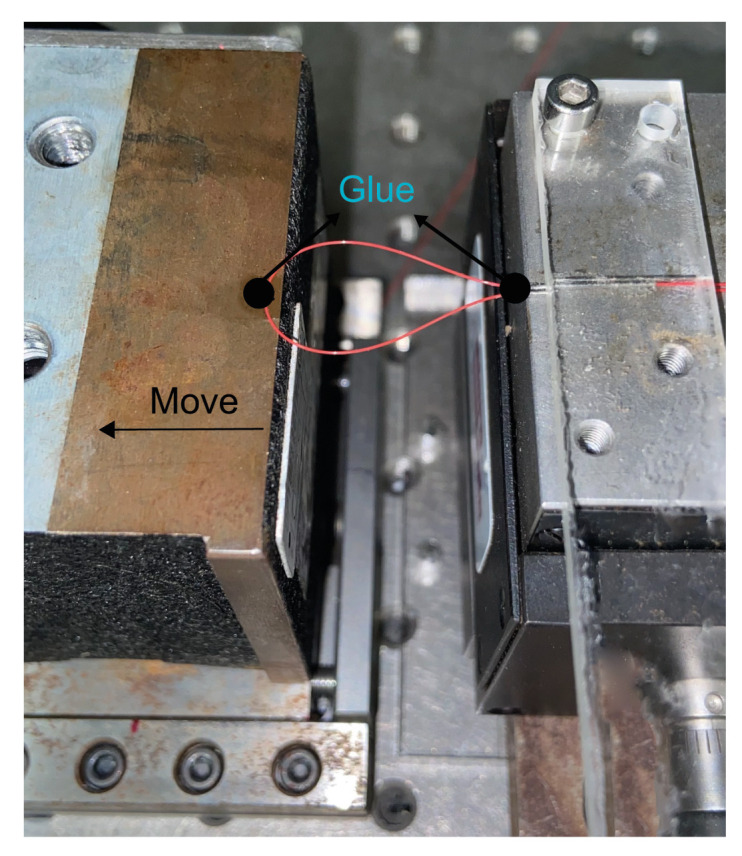
Picture of the proposed setup to micro-displacement analysis.

Before the characterization using a 3D piece, the sensor was submitted for micro-displacement characterization. The fiber sensor was fixed between two platforms, as depicted in Figure 3. Two values of radius of curvature were chosen: 5 and 6 mm. Although the bending radius of 4 mm presents an optimum interference effect, it has a spectral part of nearly the noise and, due to this, it was not chosen. These sensors, with 5 and 6 mm of radius, were submitted to a wavelength shift analysis. For the optical power transmitted analysis, a radius of 9 mm was chosen. This radius was the one that fitted the piece better than the 10 mm.

**Figure 4:**
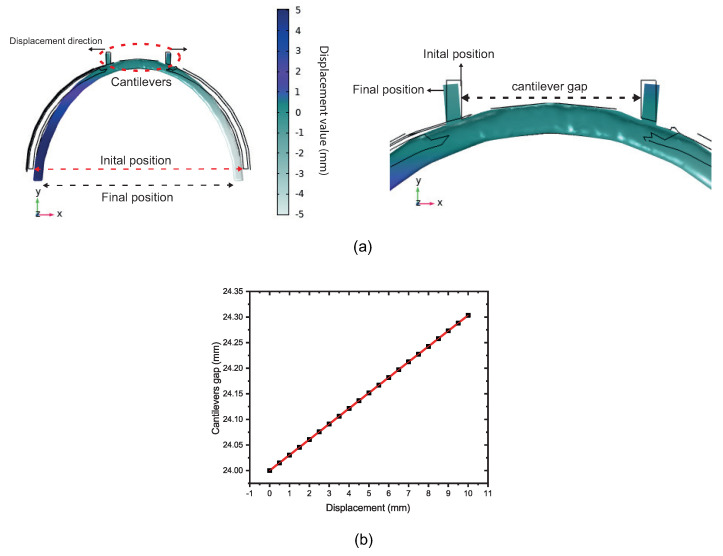
Static structural analysis of the piece with cantilevers based on FEM. (**a**) Schematic of the mechanism to increase the cantilevers gap in the 3D piece. (**b**) The linear relationship between cantilevers gap and displacement applied in the 3D piece.

**Figure 5:**
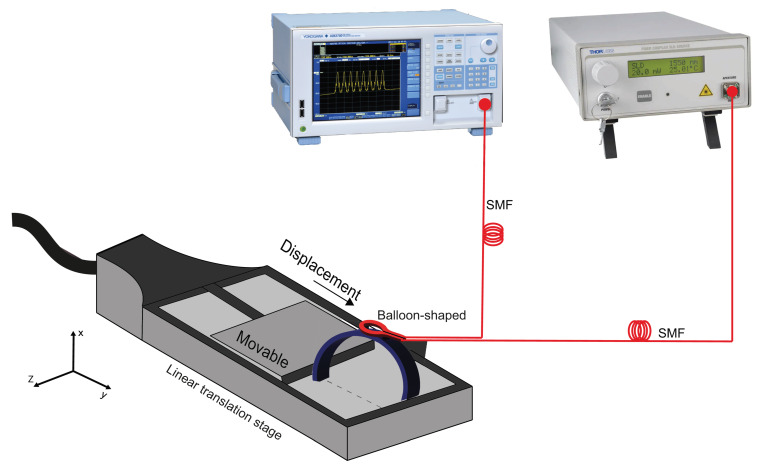
Schematic of the proposed experimental setup.

**Figure 6:**
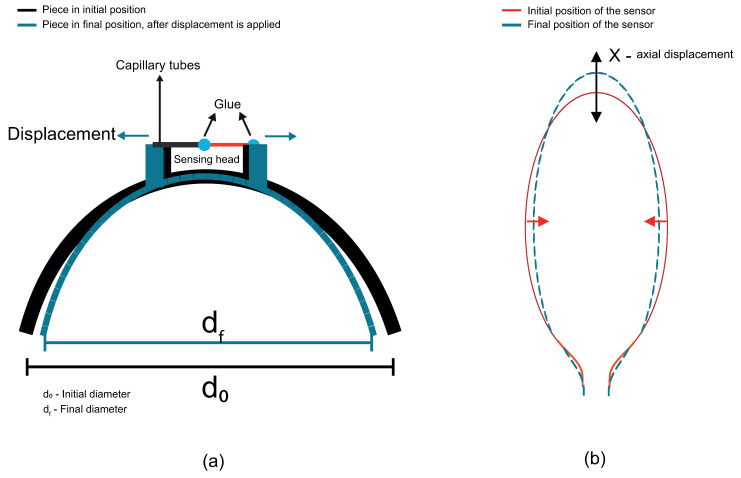
(**a**) Mechanism of operation of the 3D piece. (**b**) The axial strain direction that the optical fiber is submitted to displacement using the 3D piece.

The sensor structure is superimposed on a 3D piece. The strain gauge simulation consists of using the finite element method to help find the position of the cantilevers that would have the best offset and response to the displacement applied in the 3D piece diameter, as illustrated in Figure 4a. The cantilevers allow an increased distance between them when the diameter structure is reduced, as depicted in Figure 4a. This research revealed how widely apart the cantilevers would be and whether this would be sufficient to affect the dimensions of the balloon interferometer when it was placed on top of the cantilevers. It is possible to observe that the cantilevers suffer a shift between them, in the oval region highlighted in dotted red line in Figure 4a. It means that when the 3D printer piece is submitted to a diameter modification, the balloon suffers a dimension change due to the displacement between the cantilevers. The sensing head is fixed in such a way that the fiber is shifted due to the increased gap between the two cantilevers, as shown in Figure 4a. The setup shown in Figure 3 was used to analyze the sensor’s response to the micro-displacement. It was done because the mechanism of the 3D piece presents a micro-displacement between cantilevers. We rely on the setup data in Figure 3 to use the setup shown in Figure 4a. Figure 4b shows the linear increase in the gap as a function of the displacement applied to the 3D piece. A linear translation stage is used to apply displacement, i.e., a change in diameter, and therefore cause the change in the sensing head dimension, as illustrated in Figure 5. With regard to the displacement detection, it is possible to make a correlation with the radius change of the balloon-shaped interferometer. Figure 6a shows the schematic diagram of the displacement sensor. The 3D piece material chosen was Polylactic Acid. This material is widely utilized in the production of structures fabricated with the aid of 3D printers.

Figure 6a shows the axial strain direction that the optical fiber is subjected to using the 3D piece. The blue arrows illustrate that the sensing head is strained in opposite directions, that is, the increasing distance between cantilevers. Consequently, the sensor presents a change in their radius of curvature, leading to an optical power and wavelength change. When an axial displacement (x) is applied to the 3D structure and then the diameter is decreased, the bending diameter of the balloon-shaped interferometer is changed as well, as depicted in Figure 6b. According to Equations (3) and (5), the change in the radius of curvature will lead to a change in the effective refractive index difference Δn, which will lead to a change in $\lambda_{d}$ [20].

## 4. Results and Discussions

The results were divided into two subsections. At first, the proposed balloon-like interferometer was submitted just for micro-displacement (without a 3D piece). This test was performed to analyze the response to the micro-displacement between the cantilever distances. After the micro-displacement analysis, the proposed prototype was submitted for displacement. The second dip resonance, as depicted in Figure 2c, was used in all experiments shown in this section for the wavelength analysis due to being the dip that presented linearity in the results.

The experiments using radius values of 5 and 6 mm were performed since the radii presented very well-defined dips. After that, it was possible to observe that the sensor with a 5 mm showed the best sensitivity to micro-displacement. For the intensity analysis, we decided to choose the sensor with a 9 mm after analyzing the spectrum that can be seen in Figure 2c, in the paper. It was possible to observe that at a radius of 9 mm, there is no resonance interference very well defined and, due to it, the intensity analysis can be better.

### 4.1. Micro-Displacement Characterization

**Figure 7:**
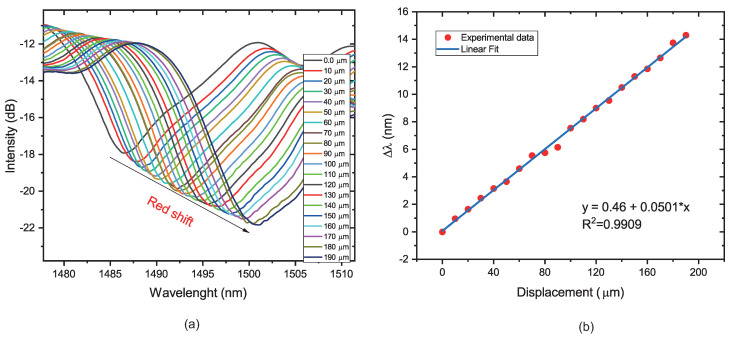
(**a**) Transmission spectra response for 5 mm of radius of curvature. (**b**) Dip wavelength changes as a function of the micro-displacement applied.

**Figure 8:**
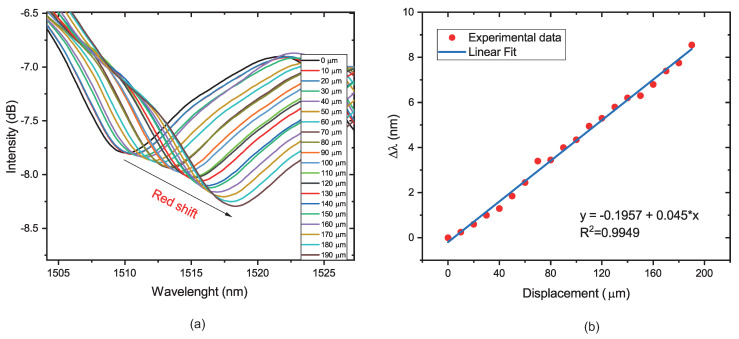
(**a**) Transmission spectra response for 6 mm of radius of curvature. (**b**) Dip wavelength changes as a function of the micro-displacement applied.

To characterize the response of the sensor to the micro-displacement, the fiber sensor was fixed between two platforms. Then, the distance was increased from 0 to 190 μm with a step of 10 μm. Based on the balloon-like interferometer formation, the radius values chosen were 5 and 6 mm. The output transmission spectra were recorded at each measurement point, as shown in Figure 7a and Figure 8a. The position of the dip wavelength may be seen to exhibit a considerable red-shift as the distance between platforms grows, which is caused by a change in the balloon-shaped object’s radius of curvature for both radii values, as illustrated in Figure 7b and Figure 8b. The sensitivity of the sensor with a radius of 5 mm was calculated to be 74.3 pm/μm, and the sensor sensitivity with a radius of 6 mm was calculated to be 45 pm/μm. Regarding the linearity, the sensors present good values of $R^{2}$, 0.9909, and 0.9949, respectively. It is possible to observe a wavelength shift when the radius of curvature is reduced from 6 to 5 mm. In addition, the sensor presents an intensity modulation when the displacement is increased. This is due to the greater evanescent field, which is sensitive to changes in the external RI, present in the lower radius of curvature [19,41].

### 4.2. Proposed Optical Strain Gauge

For the test with the proposed optical strain gauge, it was chosen the interferometer with a radius of 5 mm and 9 mm, based on the balloon-like interferometer formation and micro-displacement characterization. Firstly, the wavelength shift sensitivity of the proposed sensor was investigated using a radius of 5 mm. Finally, the interferometer with a radius of 9 mm was investigated for intensity variation. For both cases, the tests were made with a constant temperature due to a temperature sensitivity described in the literature [21,34,36,39,40].

#### 4.2.1. Wavelength Shift

**Figure 9:**
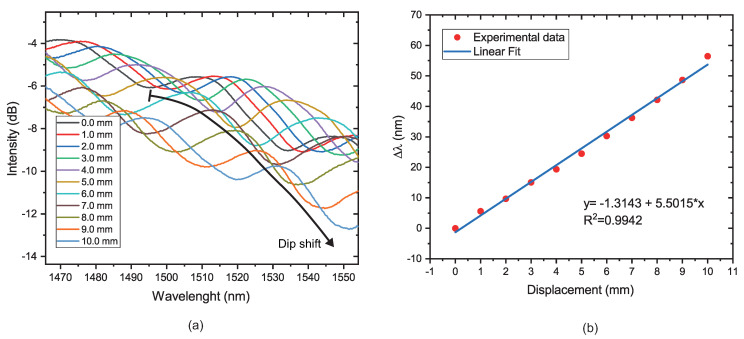
(**a**) Transmission spectra of the proposed prototype. (**b**) The relationship between displacement applied in the 3D piece and resonant wavelength.

In this experiment, the response of the proposed prototype with a radius of 5 mm was investigated. The prototype is submitted to displacement with the aid of a linear translation stage; see Figure 5. Then, the displacement is applied from 0 to 10 mm with a step of 1 mm, i.e., the diameter of the 3D piece is reduced from 90 mm to 80 mm. The transmission spectra of the sensor for different displacements applied in the 3D piece are measured and shown in Figure 9a. It can be seen that with the displacement increasing, the position of the dip wavelength shows a redshift. The black arrow at 1494.7269 nm is the first dip position used for the wavelength shift analysis. A black arrow that marks the beginning of the dip and the point at which the wavelength shift ends has been added to make it simpler to locate. The sensitivity obtained of the proposed prototype is 55.014 nm for a displacement measurement range from 0 to 10 mm. In addition, it is possible to verify the sensitivity to strain. For this case, the sensitivity is 500.14 pm/μϵ. The linear relationship between displacement (or strain) and the resonant wavelength is shown in Figure 9b, where $R^{2}$ is equal to 0.9942. The experimental results confirmed the previously discussed theoretical analysis.

Table 1 depicts a comparison between the sensor configuration developed in this work with three sensors used to measure the same parameter. It is possible to observe that the proposed structure in this work presents a greater sensitivity and dynamic range.

#### 4.2.2. Transmitted Optical Power

Another possibility is to measure the displacement by means of the transmitted optical power instead of the wavelength analysis. In this way, it is not necessary to analyze the variation in the resonance peaks or dips, but rather the transmission loss. For this reason, it was decided to use a sensor head diameter of 9 mm. Then, with the reduction of the diameter of the 3D piece, the distance between the cantilevers increases, which introduces a loss in the optical signal. This loss is a function of the displacement.

**Figure 10:**
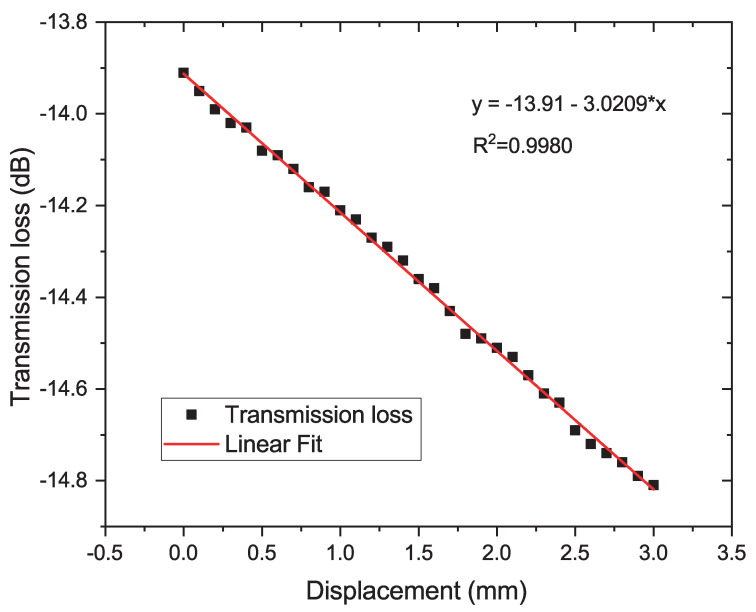
Transmitted intensity of the balloon-shaped interferometer as a function of the 3D piece displacement.

For this experiment, a radius of 9 mm was used because there is no resonance interference. There is an increase in the gap between the cantilevers as the diameter of the 3D piece is reduced. The linearity of the transmission loss as a function of the 3D piece displacement is excellent with a sensitivity of −0.0003 dB/μm (or −0.3 dB/mm) in the dynamic range from 0 to 3 mm with a step of 10 μm, as depicted in Figure 10. In this case, the dynamic range was chosen to be 3 mm because the objective of this experiment was to verify small variation with displacement steps of 10 μm.

## 5. Conclusions

In this research, it is suggested an experimental study of the straightforward process is based on the balloon-like interferometer and additive manufacturing acting as an optical strain gauge. The examination of wavelength change and intensity data shows that the displacement can be measured using the configuration proposed in this work. The main advantages of the proposed sensor are that it is simple and fast to produce and that it has a low cost. Other advantages of the sensor over standard strain gauges include electromagnetic immunity and tolerance to conditions in harsh environments, such as corrosion and high humidity. To optimize the process parameters, the mechanical behavior and micro-displacement response of the sensing head were investigated. The experimental results show a high sensitivity of 500.14 pm/μϵ and a great linearity of 0.9942 in the range from 0 to 10 mm of displacement. This dynamic range can be used for a variety of opportunities in the real world in numerous disciplines, such as civil engineering, pipelines, trunk growth, and so on. Additionally, the use of additive manufacturing offers the chance to employ biodegradable thermoplastics with high strength and high elastic modulus to obtain the shape that suits the sensor head the best. Due to it, the balloon-like interferometer’s dynamic range can be increased. Finally, it is possible to use a simple power meter to interrogate the sensing head when a radius of curvature of 9 mm is used.

## Figures and Tables

**Table 1:** Comparison of this work results to other works related in literature.

Structure	Sensitivity (pm/μϵ)	Dynamic Range (μm)	Reference
Balloon-like interferometer combined with AM	500.14	10,000	This work
FBG combined with AM	59.09	6000	[12]
FBG combined with AM	6.2	3.5	[16]
Balloon-like interferometer	180	70	[21]

## References

[1] Huang Y.H., Liu L., Sham F.C., Chan Y.S., Ng S.P. (2010). Optical strain gauge vs. traditional strain gauges for concrete elasticity modulus determination. Optik.

[2] Marek H., Nezich N., Jing K., Mario H. (2012). A Novel Class of Strain Gauges Based on Layered Percolative Films of 2D Materials. Nano Lett..

[3] Bolton W. (2021). Chapter 2—Instrumentation System Elements. Instrumentation and Control Systems.

[4] Siren K., Rosén G., Vad J., Nielsen P.V., Goodfellow H., Tähti E. (2001). 12—Experimental Techniques. Industrial Ventilation Design Guidebook.

[5] Raffaella D.S. (2015). Fibre Optic Sensors for Structural Health Monitoring of Aircraft Composite Structures: Recent Advances and Applications. Sensors.

[6] Du Y., Chen Y., Zhu C., Zhuang Y., Huang J. (2017). An embeddable optical strain gauge based on a buckled beam. Rev. Sci. Instrum..

[7] Leal-Junior A.G., Diaz C.A., Avellar L.M., Pontes M.J., Marques C., Frizera A. (2019). Polymer optical fiber sensors in healthcare applications: A comprehensive review. Sensors.

[8] Qian Y., Zhao Y., Wu Q.L., Yang Y. (2018). Review of salinity measurement technology based on optical fiber sensor. Sens. Actuators B Chem..

[9] Zhou X., Li X., Li S., An G.W., Cheng T. (2018). Magnetic field sensing based on SPR optical fiber sensor interacting with magnetic fluid. IEEE Trans. Instrum. Meas..

[10] Wang Z., Hua S., Wang D., Xu W., Yang S. (2019). Design and verification of FBG strain gauge. J. Eng..

[11] Hu Q., Wang X., Guan M., Wu B. (2019). Strain Responses of Superconducting Magnets Based on Embedded Polymer-FBG and Cryogenic Resistance Strain Gauge Measurements. IEEE Trans. Appl. Supercond..

[12] Cardoso V.H., Caldas P., Giraldi M.T.R., Frazao O., de Carvalho C.J.R., Costa J.C., Santos J.L. (2021). Experimental investigation of a strain gauge sensor based on Fiber Bragg Grating for diameter measurement. Opt. Fiber Technol..

[13] Abdi A.M., Kost A.R. (2007). A fiber optic, Fabry–Perot strain gauge calibrator. Smart Mater. Struct..

[14] Cardoso V.H.R., Caldas P., Giraldi M.T.R., Fernandes C.S., Frazão O., Costa J.C.W.A., Santos J.L. (2022). A Simple Optical Sensor Based on Multimodal Interference Superimposed on Additive Manufacturing for Diameter Measurement. Sensors.

[15] Unnikrishnan S., Razil M., Benny J., Varghese S., Hari C. LPG monitoring and leakage detection system. Proceedings of the 2017 International Conference on Wireless Communications, Signal Processing and Networking (WiSPNET).

[16] Li R., Chen Y., Tan Y., Zhou Z., Li T., Mao J. (2018). Sensors | Free Full-Text | Sensitivity Enhancement of FBG-Based Strain Sensor. Sensors.

[17] Zhou Z., Tan Y., Liu M., Yang W., Li Z. (2013). Actualities and development on dynamic monitoring and diagnosis with distributed fiber Bragg grating in mechanical systems. J. Mech. Eng..

[18] Wang W., Yiu H.H.P., Li W.J., Roy V.A.L. (2021). The Principle and Architectures of Optical Stress Sensors and the Progress on the Development of Microbend Optical Sensors. Adv. Opt. Mater..

[19] Zhu L., Lin Q., Yao K., Zhao N., Yang P., Jiang Z. (2021). Fiber Vector Magnetometer Based on Balloon-Like Fiber Structure and Magnetic Fluid. IEEE Trans. Instrum. Meas..

[20] Wu Y., Xiao S., Xu Y., Shen Y., Jiang Y., Jin W., Yang Y. (2018). Highly sensitive force sensor based on balloon-like interferometer. Opt. Laser Technol..

[21] Wu Y., Meng F., Li H., Yan G., Zhu L. (2019). Simultaneous measurement of micro-displacement and temperature based on balloon-like interferometer and fiber Bragg grating. Optik.

[22] Yang B., Niu Y., Yang B., Hu Y., Dai L., Yin Y., Ding M. (2018). High sensitivity balloon-like refractometric sensor based on singlemode-tapered multimode-singlemode fiber. Sens. Actuat. A Phys..

[23] Zhao Y., Liu X., Lv R.Q., Wang Q. (2017). Simultaneous measurement of RI and temperature based on the combination of Sagnac loop mirror and balloon-like interferometer. Sens. Actuat. B Chem..

[24] Tong R.J., Zhao Y., Chen M.Q., Hu X.G., Yang Y. (2019). Simultaneous measurement of RH and temperature based on FBG and balloon-like sensing structure with inner embedded up-tapered MZI. Measurement.

[25] Al-Janabi D.I., Salman A.M., Al-Janabi A. (2020). High-sensitivity balloon-like thermometric sensor based on bent single-mode fiber. Meas. Sci. Technol..

[26] Al-Hayali S.K., Salman A.M., Al-Janabi A.H. (2021). High sensitivity balloon-like interferometric optical fiber humidity sensor based on tuning gold nanoparticles coating thickness. Measurement.

[27] He X.L., Wang Z.Q., Wang D.H., Wang X.B., Liu Y., Jiang F.C., Yuan L.B. (2019). Optical Fiber Sensor for Strain Monitoring of Metallic Device Produced by Very High-Power Ultrasonic Additive Manufacturing. IEEE Sens. J..

[28] Camposeo A., Persano L., Farsari M., Pisignano D. (2019). Additive Manufacturing: Applications and Directions in Photonics and Optoelectronics. Adv. Opt. Mater..

[29] Hong C., Zhang Y., Borana L. (2019). Design, Fabrication and Testing of a 3D Printed FBG Pressure Sensor. IEEE Acess.

[30] Hong C., Zhang Y., Lu Z., Yin Z. (2019). A FBG Tilt Sensor Fabricated Using 3D Printing Technique for Monitoring Ground Movement. IEEE Sens. J..

[31] Xu Y., Wu X., Guo X., Kong B., Zhang M., Qian X., Mi S., Sun W. (2017). The Boom in 3D-Printed Sensor Technology. Sensors.

[32] Lambert A., Valiulis S., Cheng Q. (2018). Advances in Optical Sensing and Bioanalysis Enabled by 3D Printing. ACS Sens..

[33] Bourell D., Kruth J.P., Leu M., Levy G., Rosen D., Beese A.M., Clare A. (2017). Materials for additive manufacturing. CIRP Ann..

[34] Liu X., Zhao Y., Lv R.Q., Wang Q. (2016). High Sensitivity Balloon-Like Interferometer for Refractive Index and Temperature Measurement. IEEE Photonics Technol. Lett..

[35] Wu Y., Pei L., Jin W., Jiang Y., Yang Y., Shen Y., Jian S. (2017). Highly sensitive curvature sensor based on asymmetrical twin core fiber and multimode fiber. Opt. Laser Technol..

[36] Zhao L., Liu B., Wu Y., Sun T., Mao Y., Nan T. (2019). Measurement of refractive index and temperature using balloon- shaped Mach-Zehnder interferometer. Optik.

[37] Fu H., Jiang Y., Ding J., Zhang J. (2017). Low Temperature Cross-Sensitivity Humidity Sensor Based on a U-Shaped Microfiber Interferometer. IEEE Sens. J..

[38] Liu X., Zhao Y., Lv R.Q., Wang Q. (2016). Enhancement of RI Sensitivity Through Bending a Tapered-SMF-Based Balloon-Like Interferometer. J. Light. Technol..

[39] Tiana K., Wanga R., Zhanga M., Wangc X., Wanga X., Jinb G., Lewisd E., Farrelle G., Wanga P. (2020). Simultaneous measurement of displacement and temperature based on two cascaded balloon-like bent fibre structures. Opt. Fiber Technol..

[40] Ge J., Zhang Y., Zhang W., Kong L.X., Li Z., Yu L., Zhao X.L., Yan T.Y. (2020). Simultaneous Measurement of RI and Temperature Based on Compact U-Shaped Interferometer. IEEE Sens. J..

[41] Zhang J., Li Y., Yao G. (2022). High Sensitivity Balloon-Like Sensor Based on Twin-Core and Twin-Hole Fiber. IEEE Photon. J..

